# First principles calculations of the structural, electronic, magnetic, and thermodynamic properties of the Nd_2_MgGe_2_ and Gd_2_MgGe_2_ intermetallic compounds

**DOI:** 10.1038/s41598-021-89042-5

**Published:** 2021-05-25

**Authors:** S. Menouer, O. Miloud Abid, A. Benzair, A. Yakoubi, H. Khachai, U. Schwingenschlögl

**Affiliations:** 1 Laboratoire d’Étude des Matériaux et Instrumentations Optiques, Département Matériaux et Développement Durable, Faculté des Sciences Exactes, Université Djillali Liabès de Sidi Bel Abbès, 22000 Sidi Bel Abbès, Algérie; 2 Laboratoire de Modélisation et Simulation Multi-échelle, Faculté des Sciences Exactes, Département de Physique, Université Djillali Liabès de Sidi Bel Abbès, 22000 Sidi Bel Abbès, Algérie; 3 Physical Science and Engineering Division (PSE), King Abdullah University of Science and Technology (KAUST), Thuwal, 23955-6900 Saudi Arabia

**Keywords:** Chemistry, Materials science, Physics

## Abstract

In recent years the intermetallic ternary RE_2_MgGe_2_ (RE = rare earth) compounds attract interest in a variety of technological areas. We therefore investigate in the present work the structural, electronic, magnetic, and thermodynamic properties of Nd_2_MgGe_2_ and Gd_2_MgGe_2_. Spin–orbit coupling is found to play an essential role in realizing the antiferromagnetic ground state observed in experiments. Both materials show metallicity and application of a Debye-Slater model demonstrates low thermal conductivity and little effects of the RE atom on the thermodynamic behavior.

## Introduction

The examination of intermetallic compounds with Mo_2_FeB_2_ structure type (space group *P4/mbm*), as ordered variant of the U_3_Si_2_ structure type, has been the subject of many studies in recent years due to a variety of intriguing physical properties^1^. Examples reach from superconductivity to heavy fermion behavior, Kondo lattices, and magnetocalorics^2^. Well-known members of the material class are U_2_SnCo_2_ and U_2_InPt_2_, non-Fermi liquid systems on the verge of long-range magnetic ordering, and the hybridization-gap semiconductor U_2_SnRu_2_^3^. Y_1.6_Ce_0.4_InPd_2_ and Lu_1.6_Ce_0.4_InPd_2_ show heavy fermion behavior and in Ce_2_InRh_2_ the Ce atoms realize a mixed valent state with high spin fluctuation temperature^4^. Yb_2_AlSi_2_, on the other hand, shows an intermediate valent state^5^. More than 300 intermetallic compounds M_2_XT_2_ (M = rare earth or actinoid metal; X = Mg, In, Sn, Cd; T = transition metal) are known. The X and T atoms constitute planar [XT_2_] networks such that each X atom is coordinated to four T_2_ dumb-bells^6,7^. The crystal structure can be understood as packing of distorted fragments of AlB_2_ and CsCl structure types, which form a network of octahedra and trigonal bipyramids with interstitial sites favorable for H allocation. The M atoms form a planar structure with triangular motif, which, depending on the exchange interaction, can lead to magnetic frustration, as described by the two-dimensional Shastry-Sutherland Hamiltonian $H = J\sum\nolimits_{NN} {S_{i} S_{j} } + J^{\prime } \sum\nolimits_{NNN} {S_{i} S_{j} }$, where *S*_*i*_ represents the magnetic moment at site *i*^8^*.* Magnetic frustration exists when the nearest neighbor interaction *J* and next nearest neighbor interaction *J′* are both antiferromagnetic (AF)^9^. Since *J* > *J*′, nearest neighbor atoms form a network of *J-*coupled dimers that are coupled by *J′*. The ground state is a disordered spin liquid with energy gap between the singlet and triplet states or an antiferromagnet with gapless magnetic excitations. Transition is predicted for *J*′/*J* ≈ 0.6–0.7 at 0 K^10^, although symmetry arguments suggest that an intermediate state is required, such as a helical magnet or a spin density wave^11^.

Cermets with Mo_2_FeB_2_ structure type show excellent wear resistance, low friction to non-ferrous metals, and thermal expansion coefficients close to those of steel. While they are not as strong as the commonly used hard metals^12^, the mechanical properties can be improved by Cr and Ni additions. B, V, and Mn additions reduce the grain size and remarkably increase the transverse rupture strength^13^. RE_2_MgT_2_ (RE = rare earth) compounds are used for lightweight construction in the automobile and aerospace industries^14^. They show high corrosion resistance and thus have been investigated intensively with respect to their microstructure and mechanical properties^15^. The compounds play an essential role as bio-compatible materials for healing or replacing natural bone^16^. They are also able to absorb large amounts of H, up to 8 atoms per formula unit, which weakens the magnetism, as the RE–RE exchange interaction and magnetic coupling via the conduction electrons are suppressed (while for U_2_MgT_2_ the upper limit of H absorption is 2 atoms per formula unit and hydrogenation enhances the magnetism)^17,18^. Interstitial doping with H atoms, which induces internal pressure and/or modifies the bonding between the other atoms, can be used to tune the crystal and electronic structures, particularly the magnetism, which depends critically on details of the hybridization and charge localization^19^.

M_2_XT_2_ compounds can be magnetocaloric with field-induced magnetic and/or structural transitions, where for M = RE the magnetic structure is determined mainly by the (i) RKKY (Ruderman, Kittel, Kasuya, and Yosida) interaction and (ii) crystalline electric field (which is responsible for magnetic phase transitions, for example, for RE = Nd)^20^. In magnetocaloric ferromagnets/antiferromagnets the entropy change during isothermal magnetization is negative/positive (termed positive/negative magnetocaloric effect). A positive magnetocaloric effect is interesting for magnetic refrigerators and a negative magnetocaloric effect for heat pumps^21^.

In U_2_SnT_2_ compounds (non-magnetic or magnetic depending on T) the interatomic distance between U and T increases from U_2_SnFe_2_ to U_2_SnCo_2_ and U_2_SnNi_2_, even though the atomic radius decreases from Fe (1.27 Å) to Co (1.25 Å) and Ni (1.24 Å), and the spin–orbit coupling plays an important role for the electronic states^22^. While in U_2_SnNi_2_ the *a* lattice parameter increases with the temperature and the *c* lattice parameter decreases, in the cases of U_2_SnCo_2_ and U_2_SnPd_2_ both lattice parameters increase^22^. U_2_SnNi_2_ shows AF ordering below 25 K with the U moments aligned parallel to the *c-*axis^23^. The band width of the U 5f. states decreases for heavier T = Co, Ni, Rh, Pd, Ir, and Pt^24^. Giant magnetoresistance is predicted for U_2_SnPd_2_ and U_2_InPd_2_^25^, and specific heat data classify single crystalline U_2_SnCo_2_ and U_2_InPt_2_ as non-Fermi-liquid materials^26^. Enhanced hybridization between the U 5f. and Ni 3*d* states leads in U_2_Sn(Ni_1-*x*_Co_*x*_)_2_ solid solutions for increasing *x* to a loss of AF ordering at *x* = 0.3 and in U_2_Sn(Ni_1-*x*_Pd_*x*_)_2_ solid solutions to a decrease of the Neel temperature (*T*_*N*_) up to *x* = 0.3, which is reproduced by the RKKY model^22^. At 1.5 K the U magnetic moments switch from in-plane ordering in U_2_SnPd_2_ (2 μ_B_) to out-of-plane ordering in U_2_Sn_0.65_Pd_2.35_ (0.9 μ_B_) due to enhanced hybridization between the U 5f. and Pd 4*d* states (reduced U-Pd distance)^22^.

The magnetic behavior of the Ce_2_InT_2_ compounds is determined by the filling of the T *d* bands, with Ce being trivalent in Ce_2_InCu_2_ and Ce_2_InAu_2_ but not in Ce_2_InPd_2_^27^. First principles calculations demonstrate that in Ce_2_SnPd_2_ the hybridization between the Ce 4f. and Pd 4*d* states is weak (strong localization of the Ce 4f. states and large Ce magnetic moments)^26^. Ce_2_(In/Sn)Pd_2_ alloys display transitions between AF and ferromagnetic (FM) ground states as a function of the Sn/In ratio^28,29^. Ce_2_PbPt_2_ realizes AF ordering below 3.4 K^30^. Tb_2_InCu_2_ is FM up to 81 K^29^, and Nd_2_InAu_2_ and Tb_2_InAu_2_ are ferrimagnetic and FM up to 36 and 73 K with magnetic moments of 3.5 and 9.31 μ_B_, respectively^30^. Pr_2_InNi_2_ and Nd_2_InNi_2_ undergo second order FM to paramagnetic transitions at 7.5 and 10.5 K, respectively, according to Ref. 31, whereas Ref. 32 reports Nd_2_InNi_2_ to be AF with *T*_*N*_ = 8 K. Nd_2_InNi_2_ can absorb up to 7 H atoms per formula unit at room temperature, which leads to expansion of the unit cell along the *a*- and *c*-axes and compression along the *b*-axis (orthorhombic structure with space group *Pbam*)^33^. The Nd magnetic moment of 3.55 μ_B_ resembles that of a free Nd^3+^ ion (3.62 μ_B_)^34,35^. Nd_2_InNi_2_ is an Ising antiferromagnet^36^.

Tb_2_InPd_2_ shows a significantly higher *T*_*N*_ = 32 K than Pr_2_InPd_2_ (5 K) and Nd_2_InPd_2_ (8 K)^37^, with a spin-reorientation transition at μ_0_H_c_ = 4 T. Ref. 38 confirms *T*_*N*_ = 29.4 K for Tb_2_InPd_2_. Based on neutron powder diffraction, the Tb magnetic moments of μ_eff_ = 10.54 μ_B_ (aligned along the *c*-axis) ecxeed the theoretically predicted value of 9.72 μ_B_^35,37^. Substitution of In by Pd in Ce_2_InPd_2_ results in a transition from FM to AF ordering^39^. The fact that the specific heat of Yb_2_InPd_2_ is one order of magnitude larger than that of Yb_2_InAu_2_ is due to a high Yb 4f. density of states at the Fermi level, the intermediate valence of Yb being explained by hybridization with the Pd 4*d* states^40^. RE_2_PbPd_2_ compounds with RE = Ce, Nd, Sm, Gd, Tb, Dy, Ho, Er, and Tm show AF ordering at low temperatures (*T*_*N*_ in the range of 3.6–35 K), Pr_2_PbPd_2_ is a Curie–Weiss paramagnet down to 1.72 K, and Y_2_PbPd_2_ and La_2_PbPd_2_ are weak Pauli paramagnets^41^. Ce_2_PbPd_2_ is subject to a weak Kondo effect with a Ce magnetic moment of 2.61 μ_B_, and RE magnetic moments of 4.05 and 9.85 μ_B_ are found for Nd_2_PbPd_2_ and Tb_2_PbPd_2_, respectively^41^. Ce_2_MgSi_2_ shows helical AF ordering at *T*_*N*_ = 12 K with magnetocrystalline anisotropy and a Ce magnetic moment of 2.47 μ_B_^42^. An anomaly in the electrical resistivity of Nd_2_InGe_2_ at 9 K points to AF ordering with a small FM component^43^.

In the case of the RE_2_SnNi_2_ compounds the smaller RE elements Ho, Er, Tm, Lu, and Sc result in a Mo_2_FeB_2_ structure, whereas the larger RE elements Ce, Pr, Nd, Sm, Gd, Tb, Dy, and Y result in an orthorhombic W_2_CoB_2_ structure (space group *Immm*)^44,45^. Nd_2_SnNi_2_ exhibits AF ordering below *T*_*N*_ = 21 K (which can be turned into FM ordering by a moderate magnetic field of 0.25 T) and two further magnetic transitions at 17.7 and 14–15 K^20^. Ce_2_SnNi_2_ is a Kondo system with *T*_*N*_ = 4.7 K and *T*_*K*_ ≈ 8 K, and Gd_2_SnNi_2_ and Tb_2_SnNi_2_ show oscillatory magnetocaloric effects^20^. Tb_2_SnNi_2_ transforms under high pressure (8 GPa) and high temperature (1470 K) to the Mo_2_FeB_2_ structure^44^. In the W_2_CoB_2_ structure it shows AF ordering below *T*_*N*_ = 66 K (Tb magnetic moment of 8.7 μ_B_; very close to a free Tb^3+^ ion) with magnetic transitions at 42 and 8 K (probably coexistence of FM and AF ordering), and a Curie–Weiss behavior above 80 K (Tb magnetic moment of 7.7 μ_B_)^20^. In the temperature range of 5–220 K, Nd_2_SnNi_2_ and Tb_2_SnNi_2_ do not reach their theoretical saturation magnetization in a magnetic field of 100 kOe, which may be attributed to a canted magnetic structure^20^. For the isothermal magnetic entropy change in a magnetic field of 50 kOe values of 7.2, 0.1, 4.6, and 2.8 J/kg K are reported for Nd_2_SnNi_2_, Sm_2_SnNi_2_, Gd_2_SnNi_2_, and Tb_2_SnNi_2_, respectively^20^. Tb_2_SnNi_2_ shows striking similarities to (Pr,Ca)MnO_3_, since both these compounds are subject to coexistence of AF and FM ordering at low temperature with a magnetocaloric effect that switches from negative to positive when the temperature increases^46^.

Replacing the transition metal with a main group element has a drastic effect on the RE element and thus on the magnetic properties. To give an example, *T*_*N*_ grows from 49 K in Gd_2_MgNi_2_ (additional magnetic transitions at 20*.*7 and 4*.*5 K^47^) to 150 K in Gd_2_MgGe_2_^48^. The RE_2_MgGe_2_ compounds with RE = Nd, Gd, Tb, Dy, Ho, Er, and Tm show Curie–Weiss paramagnetism at high temperature, while Y_2_MgGe_2_ and Lu_2_MgGe_2_ display Pauli-like temperature independent paramagnetism^49^. AF ordering at 14, 13, 32, 55, 24, and 14 K is found for Nd_2_MgGe_2_, Sm_2_MgGe_2_, Gd_2_MgGe_2_, Tb_2_MgGe_2_, Dy_2_MgGe_2_, and Ho_2_MgGe_2_, respectively, while Er_2_MgGe_2_ and Tm_2_MgGe_2_ do not undergo magnetic ordering at least down to 5 K^49^. Since the type of magnetic ordering and ordering temperature are determined by the atomic interactions^50^ and there is a lack of detailed understanding, the present work addresses the class of RE_2_MgGe_2_ compounds from a first principles perspective.

## Results and discussion

**Figure 1:**
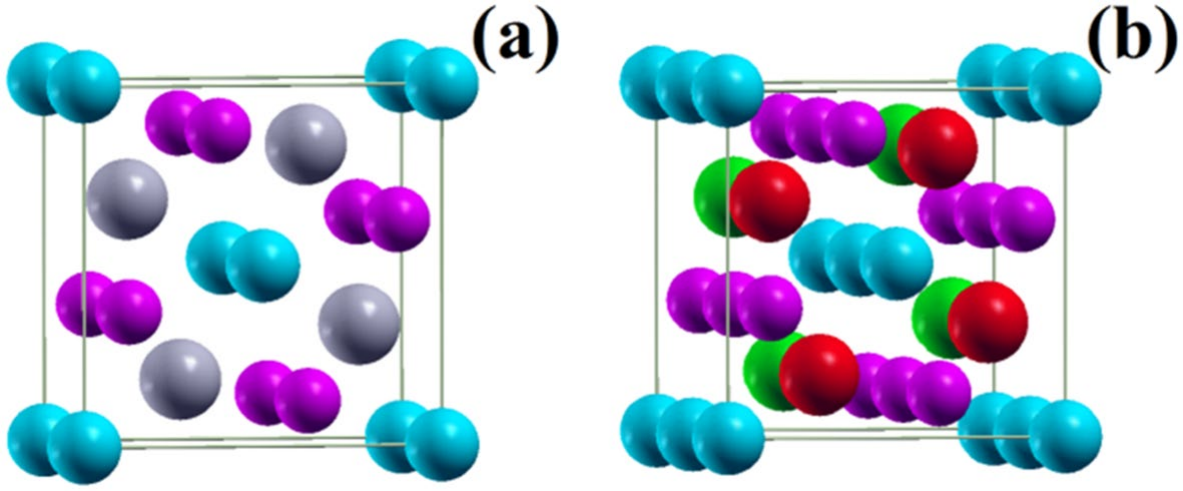
(**a**) Crystal structure. Gray, blue, and purple spheres represent the RE, Mg, and Ge atoms, respectively. (**b**) AF ordering of the RE^3+^ ions (green spheres represent spin up and red spheres spin down).

We use the full potential linear augmented plane wave plus local orbitals software WIEN2k^51^, which provides highly accurate results due to the fact that it is an all-electron implementation of density functional theory. The electronic wave functions are expanded in spherical harmonics (up to *l*_max_ = 10) within non-overlapping muffin-tin spheres centered at the nuclear sites and plane waves (limited by *K*_max_ = 7/*R*_MT,min_) in the remaining space (interstitial region). The generalized gradient approximation is chosen for the exchange–correlation functional^52^. We employ 6 × 6 × 11 and 6 × 6 × 5 Monkhorst–Pack k-grids in the structural optimizations of unit cells and 1 × 1 × 2 supercells, respectively. The unit cells are non-primitive with two formula units (Fig. 1a). In the supercell calculations spin-polarization is taken into account with the RE atoms coupled ferromagnetically within the *ab*-plane and antiferromagnetically along the *c*-axis (Fig. 1b). Densities of states are calculated using a refined 10 × 10 × 9 Monkhorst–Pack k-grid and adding spin–orbit coupling by the polarized orbital method to obtain correct magnetic moments^53,54^.

**Figure 2:**
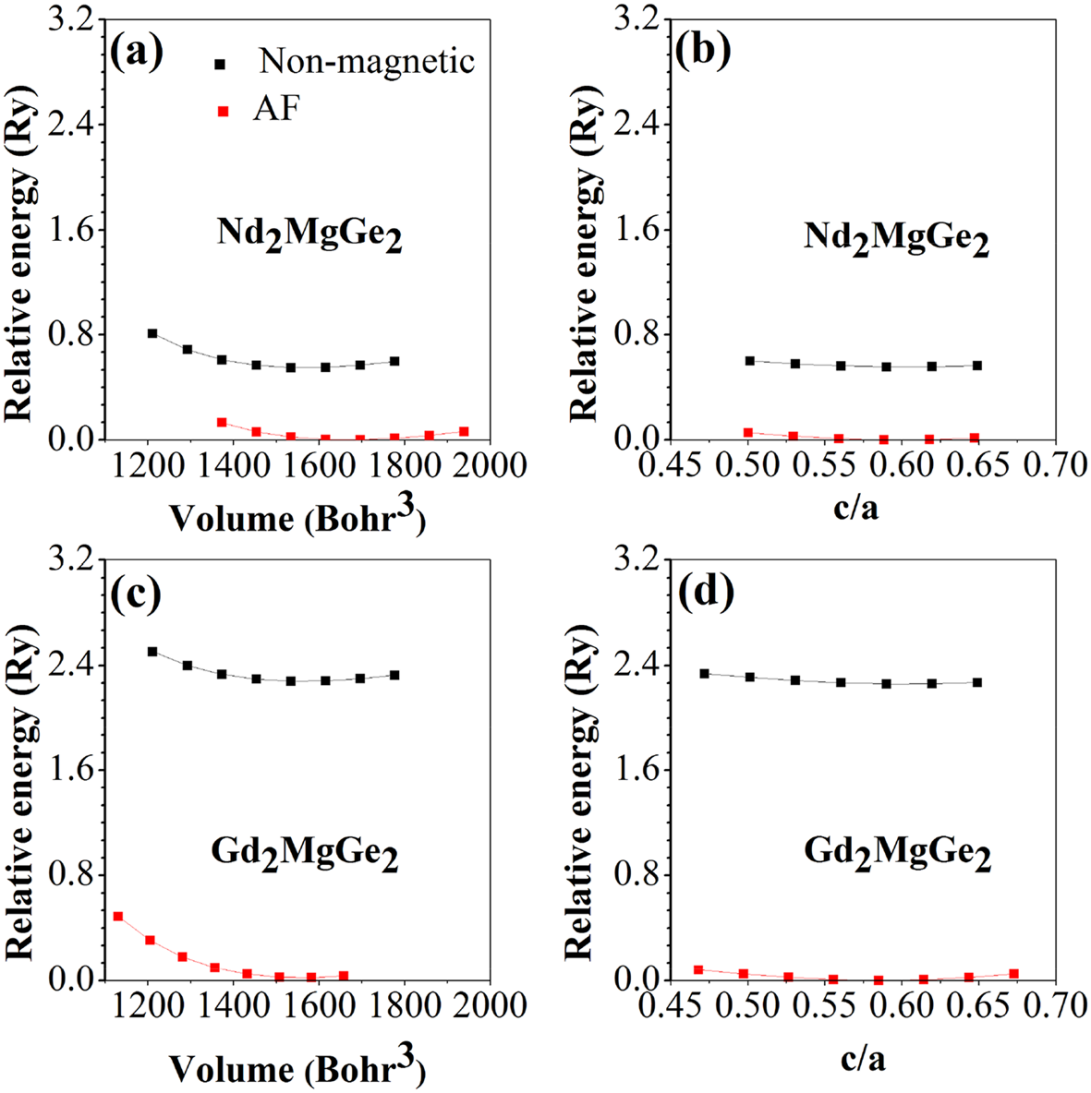
Total energy (**a**, **c**) versus volume and (**b**, **d**) versus *c*/*a* ratio.

In the tetragonal unit cell of RE_2_MgGe_2_ (Fig. 1a) the atoms occupy the Wykhoff positions RE 4 h (*x*_RE_, *x*_RE_ + 1/2, 1/2), Ge 4 g (*x*_Ge_, *x*_Ge_ + 1/2, 0), and Mg 2a (0, 0, 0). Both for non-magnetic and AF configurations, we relax *x*_RE_ and *x*_Ge_ at different volumes and fit the total energy to the Murnaghan equation of state^55^ in order to obtain the equilibrium volume (Fig. 2a,b). Then we relax *x*_RE_ and *x*_Ge_ at different *c/a* ratios and again fit the total energy to the Murnaghan equation of state in order to obtain the equilibrium *c/a* ratio (Fig. 2c,d) as well as the bulk modulus and its pressure derivative. Table 1 shows that AF ordering significantly modifies the unit cell volume for Nd_2_MgGe_2_ but not for Gd_2_MgGe_2_. It turns out that AF ordering results in energy gain of 0.5 eV per unit cell for Nd_2_MgGe_2_ and 2.3 eV per unit cell for Gd_2_MgGe_2_ with respect to the non-magnetic solution. We do not further investigate Sm_2_MgGe_2_, Tb_2_MgGe_2_, Dy_2_MgGe_2_, and Ho_2_MgGe_2_, as no qualitative difference can be expected. Y_2_MgGe_2_ and Lu_2_MgGe_2_ are not of interest, because no magnetic ordering is obtained, in agreement with Ref. 49. For Er_2_MgGe_2_ and Tm_2_MgGe_2_ our calculations predict magnetic ground states. However, absence of magnetic ordering above 5 K implies that the compounds undergo low temperature phase transitions, i.e., our results are not of experimental relevance and therefore excluded from the following discussions.

**Table 1:** Lattice parameters (*a*, *c* in Å), internal parameters (*x*_RE_, *x*_Ge_), equilibrium volume (*V*_0_ in Bohr^3^), bulk modulus at 0 K (*B* in GPa), and pressure derivative of the bulk modulus (*B*′). Experimental values from Refs.^48,49^ are given for comparison.

	Nd_2_MgGe_2_			Gd_2_MgGe_2_		
	Non-magnetic	AF	Experiment	Non-magnetic	AF	Experiment
*a*	7.28	7.43	7.41	7.28	7.32	7.29
*c*	4.37	4.46	4.36	4.36	4.30	4.28
*x*_RE_	0.176	0.177	0.178	0.174	0.177	–
*x*_Ge_	0.375	0.378	0.379	0.375	0.376	–
*V*_0_	1565	1661	1616	1561	1559	1536
*B*	62	53	–	55	70	–
*B*′	4	5	–	4	4	–

The obtained AF lattice constants in Table 1 deviate from the experimental values by less than 0.4% (*a*) and 2.3% (*c*)^48,49^. They turn out to be smaller for Gd_2_MgGe_2_ than Nd_2_MgGe_2_ though Gd is heavier than Nd (lanthanide contraction) and the shortest RE–RE distances within the *ab*-plane (3.72 Å for Nd_2_MgGe_2_, 3.66 Å for Gd_2_MgGe_2_) are significantly smaller than those along the *c*-axis (4.46 Å for Nd_2_MgGe_2_, 4.30 Å for Gd_2_MgGe_2_). This confirms the magnetic structure model of Ref. 49 that the localized RE 4f. spins realize FM ordering within the *ab*-plane and AF ordering mediated by RKKY interaction along the *c*-axis. As expected, we find *B* ∝ *V*_0_^‒1^, where *V*_0_ is the equilibrium unit cell volume. Rather small values of *B* reflect softness of the compounds under study. The magnetic moment $M = g \sqrt {J\left( { J + 1 } \right)}$ depends on the Landé factor $g = 1 + \frac{{J\left( {J + 1} \right) + S\left( {S + 1} \right) - L\left( {L + 1} \right)}}{{2J\left( {J + 1} \right)}}$ with *J* = *L* ± *S* for a less/more than half-filled shell^56^. Comparison of the obtained magnetic moments with experiment in Table 2 confirms the validity of our theoretical approach. The fact that they are almost purely of 4f. origin and close to those of free atoms demonstrates localized magnetism.

**Table 2:** Spin moment (*S*), orbital moment (*L*), total moment (*J*), Landé factor (*g*), and total magnetic moment (*M* in μ_B_). Free atom (RE^3+^)^56^ and experimental^49^ values are given for comparison.

	Nd_2_MgGe_2_			Gd_2_MgGe_2_		
	GGA+SOC+OP	Nd^3+^	Experiment	GGA+SOC+OP	Gd^3+^	Experiment
*2S*	3.1	3	–	6.8	7	–
*L*	5.6	6	–	0	0	–
*J*	4	4.5	–	3.4	3.5	–
*g*	0.7	0.7	–	2	2	–
*M*	3.1	3.6	3.9	7.7	7.9	8

**Figure 3:**
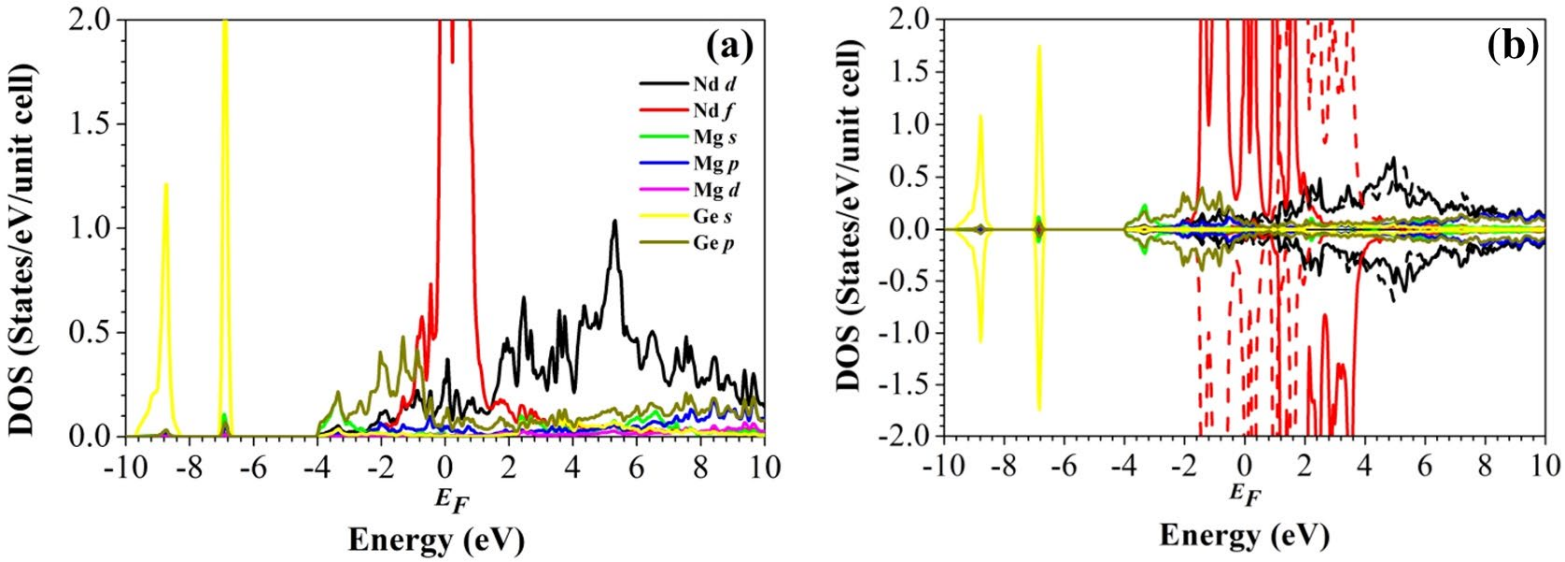
Partial densities of states of Nd_2_MgGe_2_ in the (**a**) non-magnetic and (**b**) AF configurations. Full and dashed lines distinguish between the spin sublattices.

**Figure 4:**
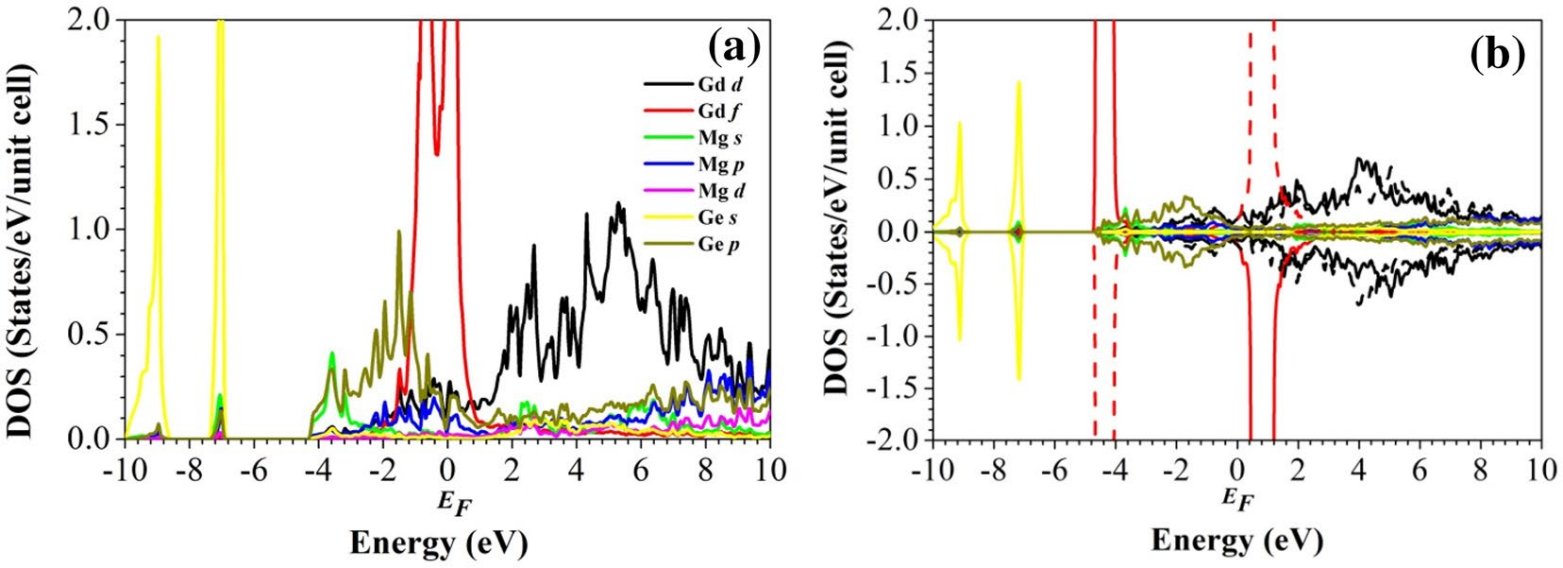
Partial densities of states of Gd_2_MgGe_2_ in the (**a**) non-magnetic and (**b**) AF configurations. Full and dashed lines distinguish between the spin sublattices.

To study the nature of the chemical bonding, we evaluate the electronic density of states (DOS) in Figs. 3 and 4. We note that the local density approximation yields qualitatively the same behavior (Figure S1 in the Supporting Information) and that the wave function expansions are well converged (Figure S2 in the Supporting Information). For both the non-magnetic and AF configurations of the two compounds we obtain similar results, in particular a finite DOS at the Fermi level, i.e., a metallic state. The high non-magnetic DOS at the Fermi level (mainly Nd/Gd 4f. states) points to magnetism according to the Stoner criterion^57^. The symmetric shape of the AF DOS is due to the absence of a total magnetic moment. The AF DOS shows strong contributions of the Ge 4* s* states at low energy, the Ge 4*p* states below the Fermi level, and the Nd/Gd 4f. states above the Fermi level. The Nd 4f. states are found from − 2 eV to 4 eV, whereas the Gd 4f. states give rise to pronounced peaks around − 4 eV and just above the Fermi level. As the compounds are isostructural, the nature of the chemical bonding is similar (except for the splitting of the Nd/Gd 4f. states) and the physical properties thus are expected to be comparable.

**Figure 5:**
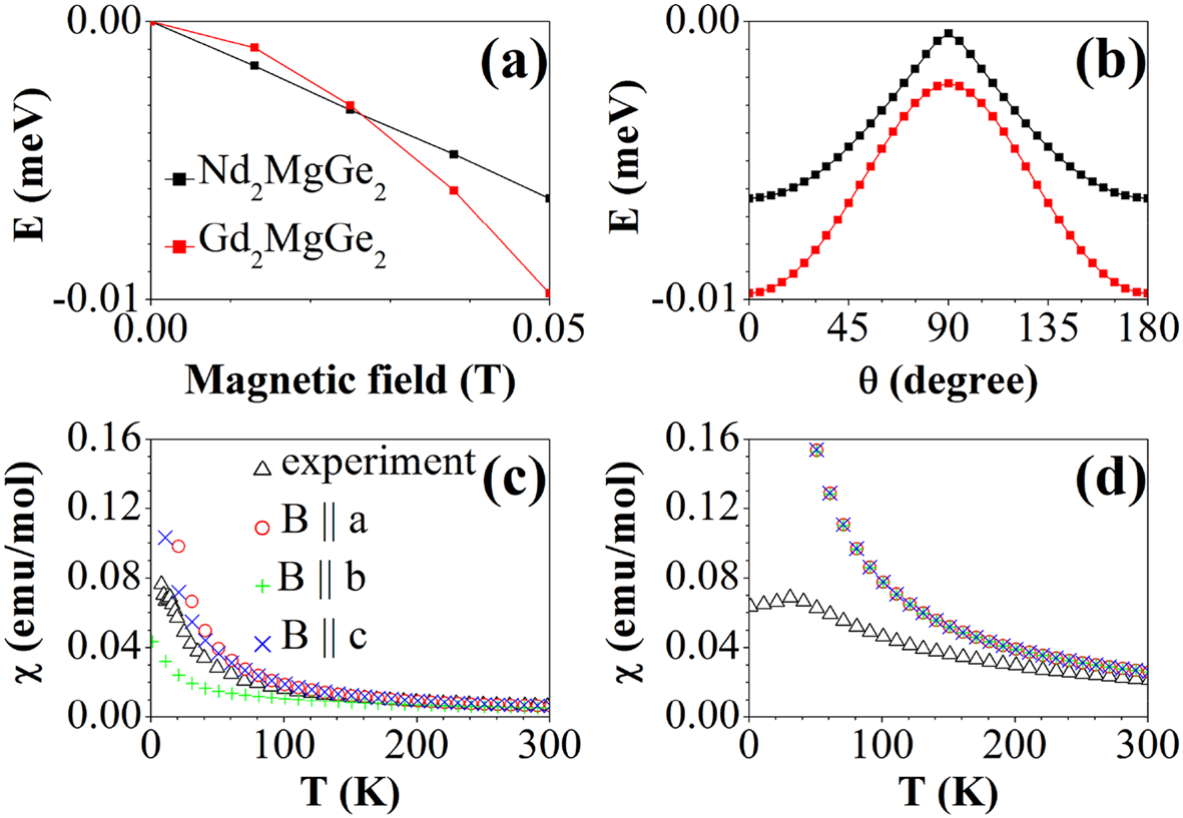
Dependence of the lowest ground state multiplet energy on the (**a**) strength of an external magnetic field within the *ab*-plane and (**b**) angle of a 0.05 T magnetic field with respect to the *a*-axis. Temperature dependence of the magnetic susceptibility for (**c**) Nd_2_MgGe_2_ and (**d**) Gd_2_MgGe_2_.

To better understand the magnetism, we evaluate as dominant perturbation the crystal field splitting of the localized and correlated 4f. states. We follow the methodology of Refs.^58–62^ to extract the crystal field parameters from our first principles calculations and obtain the ground state multiplet energies 0, 2, 13, 15, and 16 meV (first excited state: 245 meV) for the Nd^3+^ ions and 0, 0.01, 0.03, and 0.05 meV (first excited state: 3980 meV) for the Gd^3+^ ions. Nd^3+^ (4*f*^3^ electronic configuration) and Gd^3+^ (4*f*^7^ electronic configuration) are Kramer ions. While the ground state of Nd^3+^ (^4^*I*_9/2_, *S* = 3/2, *L* = 6, *J* = 9/2) is split by the crystal field into five Kramer doublets (in agreement with Ref. 63), Gd^3+^ has zero orbital moment in the ground state (^8^*S*_7/2_, *S* = 7/2, *L* = 0, *J* = 7/2) and, consequently, is not influenced by the crystal field in first approximation. The angular dependence of the ground state multiplet energies in a magnetic field aligned within the *ab*-plane (Fig. 5a,b) shows that *a* is the easy magnetic axis and *b* is the hard magnetic axis. The obtained magnetic susceptibilities approach the experimental curve for increasing temperature (Fig. 5c,d).

**Figure 6:**
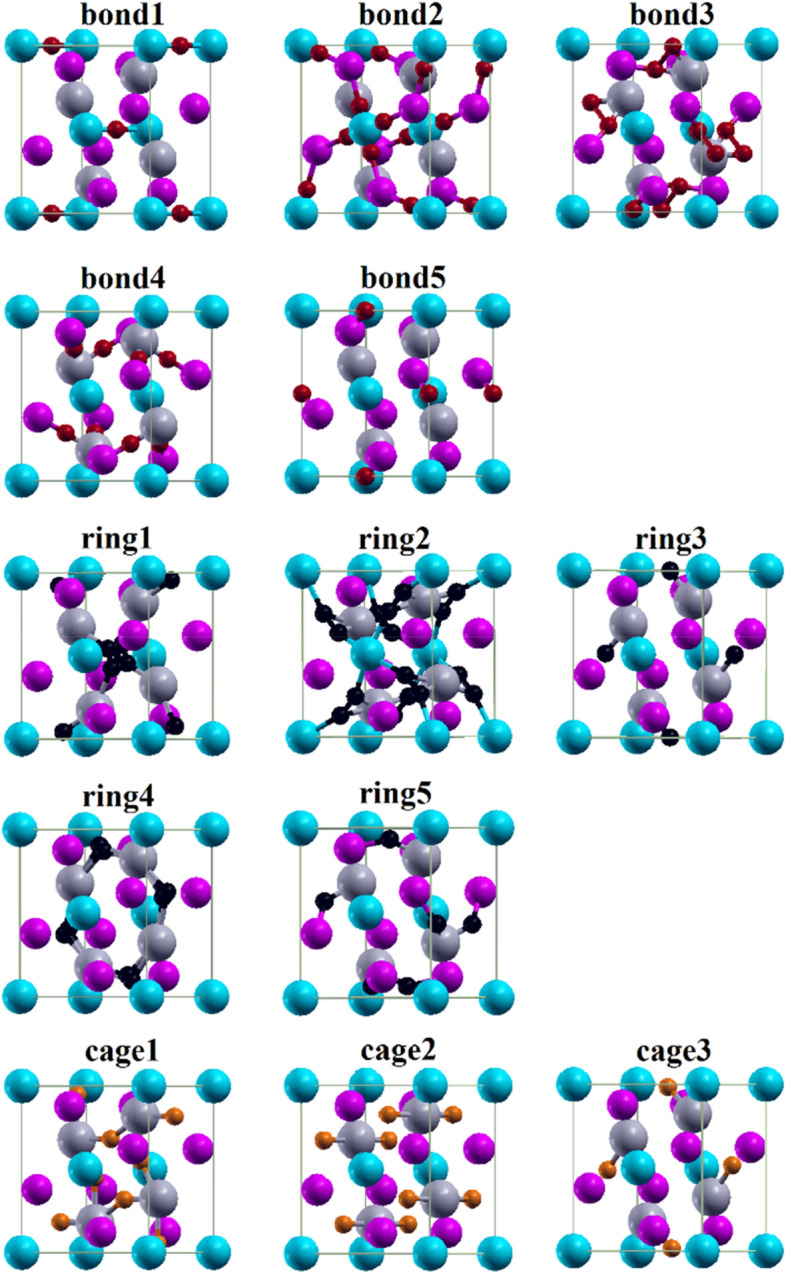
Bond CPs (red spheres), ring CPs (black spheres), and cage CPs (orange spheres). Gray, blue, and purple spheres represent the RE, Mg, and Ge atoms, respectively.

We analyze the electron density in terms of the quantum theory of atoms in molecules^64^ (CRITIC code^65^) by means of the critical points (CPs), which are categorized into nuclear, bond, ring, and cage CPs according to the Hessian matrix. Table 3 lists the locations and characteristics of all the symmetry-inequivalent CPs. As expected, the CPs are very similar for the two compounds. In Fig. 6 we represent the bond CPs (same order as in Table 3) by red spheres to identify the atoms linked by them. For Nd_2_MgGe_2_ the Ge-Mg bond CP is closer to Mg (2.03 Å) than Ge (3.41 Å), while the Ge–Nd bond CP is more centered. The electron density at the bond CPs is small and the Laplacian is close to zero (positive in the cases of the Ge-Mg and Ge–Nd bonds, negative in the cases of the Mg-Mg and Ge–Ge bonds; Table 3). For Gd_2_MgGe_2_ the Ge-Gd bond CP is closer to Gd (2.86 Å) than Ge (3.00 Å). Again the electron density at the CPs is small and the Laplacian is close to zero (positive in the case of the Ge-Gd bonds, negative in the cases of the Ge–Ge and Mg-Mg bonds; Table 3).

**Table 3:** CPs with charge density (ρ in electrons/Bohr^3^) and its Laplacian (∇^2^ρ in electrons/Bohr). The names refer to Fig. 6.

CP	Nd_2_MgGe_2_					Gd_2_MgGe_2_				
	*x*	*y*	*z*	ρ	∇^2^ρ	*x*	*y*	*z*	ρ	∇^2^ρ
Bond1	0	0	0.5	0.013	− 0.001	0	0	0.5	0.012	− 0.001
Bond2	0.045	0.140	0	0.021	0.038	0.045	0.140	0	0.021	0.038
Bond3	0.486	0.152	0.269	0.029	0.039	0.473	0.144	0.260	0.026	0.040
Bond4	0.282	0.782	0.740	0.033	0.043	0.277	0.777	0.742	0.031	0.031
Bond5	0.5	0	0	0.055	− 0.014	0.5	0	0	0.055	− 0.014
Ring1	0.044	0.925	0.5	0.012	0.003	0.051	0.920	0.5	0.011	0.003
Ring2	0.256	0.128	0.069	0.014	0.015	0.208	0.107	0.147	0.013	0.017
Ring3	0.5	0	0.5	0.014	0.031	–	–	–	–	–
Ring4	0.405	0.872	0.5	0.015	0.011	0.368	0.899	0.5	0.014	0.011
Ring5	0.431	0.069	0.769	0.024	0.044	0.430	0.070	0.774	0.024	0.037
Cage1	0.448	0.683	0.5	0.011	0.014	0.457	0.666	0.5	0.010	0.009
Cage2	0.174	0.674	0	0.013	0.012	0.175	0.675	0	0.012	0.013
Cage3	0.485	0.985	0.5	0.014	0.030	0.5	0	0.5	0.013	0.029

**Figure 7:**
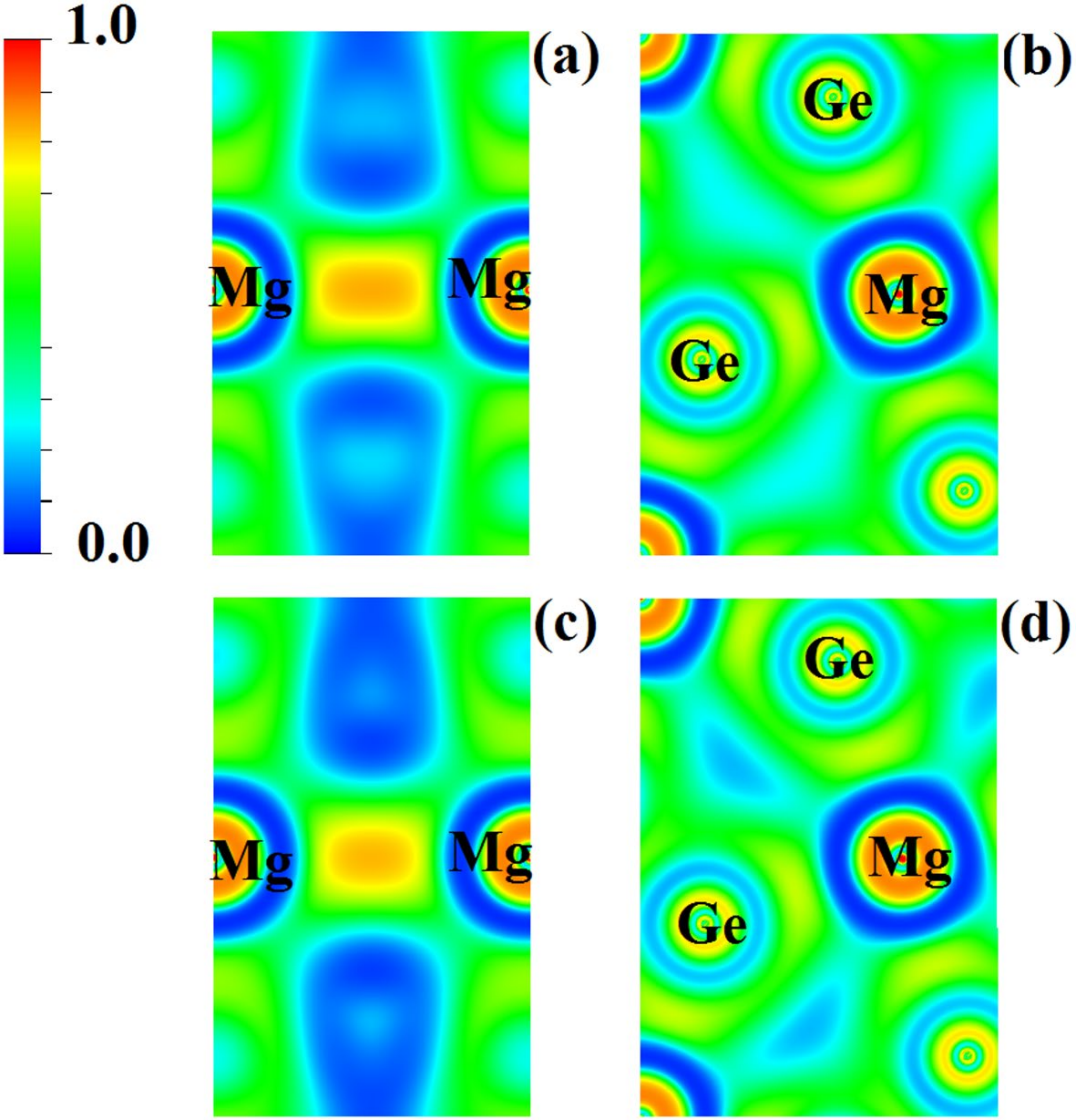
Electron localization function in the (**a**, **c**) (200) and (**b**, **d**) (001) planes for (**a**, **b**) Nd_2_MgGe_2_ and (**c**, **d**) Gd_2_MgGe_2_.

Table 4 lists the atomic volumes and corresponding integrated charges. 48% and 51% of the unit cell volume is filled by Ge for Nd_2_MgGe_2_ and Gd_2_MgGe_2_, respectively, 40% and 38% by RE, and 11% by Mg. We find a net transfer of charge from Nd/Gd and Mg to Ge, in agreement with the Pauling electronegativity. The obtained oxidation states deviate significantly from the nominal values of + 3 (Nd/Gd), + 2 (Mg), and ‒4 (Ge), confirming a metallic nature^48^, in contrast to the semiconducting charge distribution with neutral Mg proposed in Ref. 66. The global charge transfer is described by the ionicity $c = \frac{1}{N}\sum\nolimits_{i = 1}^{N} {\frac{{\Delta Q_{i} }}{{OS_{i} }}}$^67^, where *N* is the number of atomic sites *i* and $\Delta Q_{i}$/*OS*_*i*_ is the ratio of the actual and nominal oxidation states. The values of 37% and 39% for Nd_2_MgGe_2_ and Gd_2_MgGe_2_, respectively, are indicative of dominant covalent bonding. Indeed, the electron localization function demonstrates strong covalent Mg-Mg bonds (Fig. 7a,c) and the electronic charges in Table 4 in conjunction with Fig. 7b,d point to polarized covalent Mg-Ge bonds. We estimate the degree of metallicity in terms of the electron density flatness *f* = ρ_min_/ρ_max_, where ρ_min_ is the minimum electron density (at the cage1 CP) and ρ_max_ is the maximum electron density (at the bond5 CP)^65^. As *f* = 1 represents metallic bonding and *f* = 0 represents localized bonding, the obtained values of 20% and 18% for Nd_2_MgGe_2_ and Gd_2_MgGe_2_, respectively, show that metallic bonding plays a limited role.

**Table 4:** Pauling electronegativity (χ), oxidation state (Δ*Q*), and atomic volume (Ω in Bohr^3^).

Atom	Nd_2_MgGe_2_			Gd_2_MgGe_2_		
	χ	Δ*Q*	Ω	χ	Δ*Q*	Ω
Nd	1.14	0.99	158	–	–	–
Gd	–	–	–	1.20	1.03	147
Mg	1.31	0.93	89	1.31	0.98	89
Ge	2.01	− 1.46	189	2.01	− 1.53	199
Total	–	–	1564	–	–	1562

**Figure 8:**
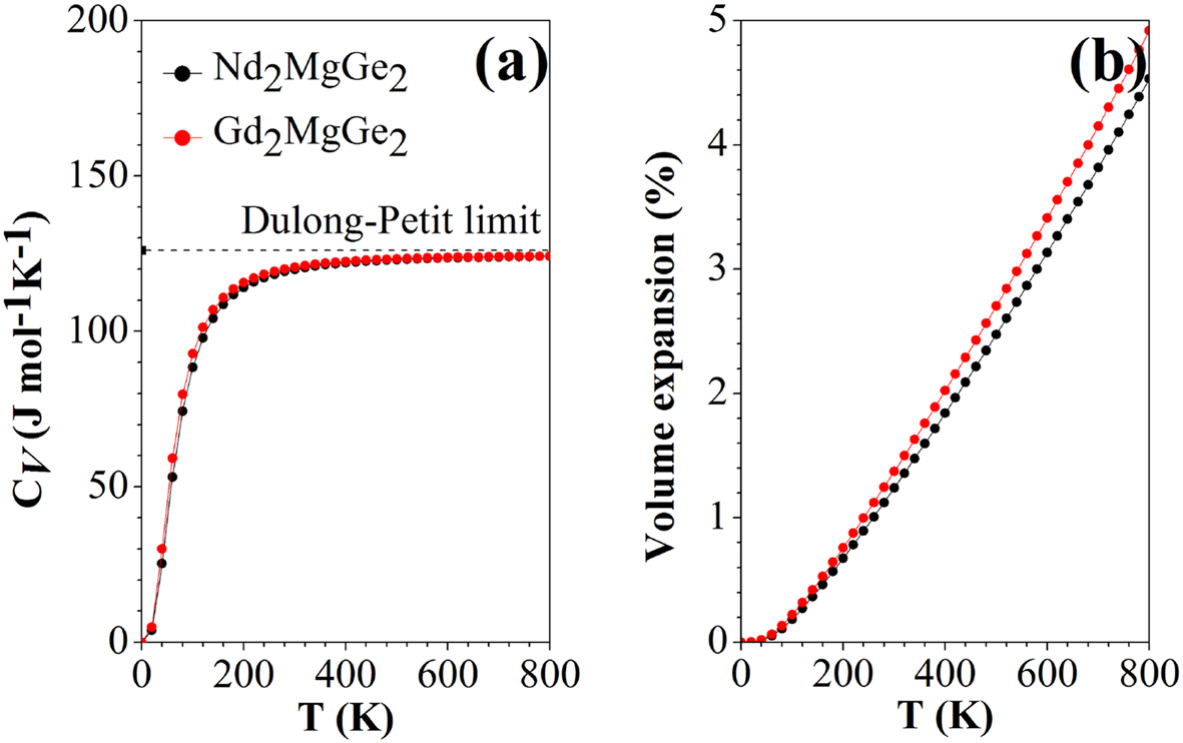
Temperature dependence of the (**a**) heat capacity and (**b**) volume expansion (unit cell).

We use a Debye-Slater model to drive the thermodynamic properties^68^. Table 5 indicates that the two compounds behave similarly although Nd_2_MgGe_2_ shows slightly larger bulk modulus and Debye temperature. The heat capacity at constant pressure (*C*_*p*_) turns out to be larger than the heat capacity at constant volume (*C*_*V*_), consistent with the relation *C*_*p*_ − *C*_*V*_ = (*α*_*V*_)^2^*BV*_*p*_*T*, where *α*_*V*_, *B*, *V*_*p*_, and *T* are the thermal expansion coefficient at constant volume, bulk modulus, volume of the primitive unit cell (Nd_2_MgGe_2_: ½ × 1592.4 Bohr^3^; Gd_2_MgGe_2_: ½ × 1590.2 Bohr^3^), and absolute temperature, respectively. According to Fig. 8a, *C*_*V*_ ∝ *T*^3^ at low temperature and the Dulong-Petit limit is approached at high temperature. Low Debye temperatures reflect low thermal conductivities and melting temperatures. According to Fig. 8b, the volume expansion starts to become linear in *T* at about 150 K.

**Table 5:** Properties at 300 K: thermal expansion coefficient (*α*_*V*_ in 10^−5^ K^−1^), vibrational contributions to the heat capacity at constant volume and pressure (*C*_*V*_ and *C*_*p*_ in J/(mol K)), isothermal and adiabatic bulk moduli (*B* and *B*_S_ in GPa), primitive unit cell volume (*V*_*p*_ in Bohr^3^), (*α*_*V*_)^2^*BV*_*P*_*T* (in J/(mol K)), and Debye temperature (*Ө*_D_ in K).

	*α*_*V*_	*C*_*V*_	*C*_*p*_	*B*	*B*_S_	*V*_*p*_	(*α*_*V*_)^2^*BV*_*p*_*T*	*Ө*_D_
Nd_2_MgGe_2_	6	120	124	57	60	796.2	4.1	266
Gd_2_MgGe_2_	6	121	125	52	53	795.1	4.3	245

## Conclusions

The structural, electronic, magnetic, and thermodynamic properties of the intermetallic compounds Nd_2_MgGe_2_ and Gd_2_MgGe_2_ have been investigated by full potential linearized augmented plane wave plus local orbitals calculations, employing the generalized gradient approximation for the exchange–correlation potential. The calculated lattice constants agree well with the available experimental data. Accounting for the spin–orbit coupling turns out to be mandatory to obtain correct magnetic moments and evaluate the electronic properties. Both compounds are found to combine metallicity with an AF ground state with localized magnetic moments. The chemical bonding turns out to be predominantly covalent. According to a Debye-Slater model, the thermal conductivity is low and the choice of the RE atom hardly affects the thermodynamic behavior.

## Supplementary Information

Supplementary Information.
