# Supplementary material for: First principles calculations of the structural, electronic, magnetic, and thermodynamic properties of the Nd2MgGe2 and Gd2MgGe2 intermetallic compounds
Source: Sci Rep. 2021 May 25;11:10870. doi: 10.1038/s41598-021-89042-5 (PMC8149671; doi:10.1038/s41598-021-89042-5)
Supplement: Supplementary file 1 — Supplementary Information. [file 41598_2021_89042_MOESM1_ESM.pdf]

Supporting Information

First principles calculations of the structural, electronic, magnetic, and thermodynamic properties of the Nd_2MgGe_2 and Gd_2MgGe_2 intermetallic compounds

S. Menouer[1], O. Miloud Abid[1], A. Benzair[2], A. Yakoubi[1], H. Khachai[1], U. Schwingenschlögl[3]

[1]*Laboratoire d'Étude des Matériaux et Instrumentations Optiques, Département Matériaux et Développement Durable, Faculté des Sciences Exactes, Université Djillali Liabès de Sidi Bel Abbès 22000, Algérie*

[2]*Laboratoire de Modélisation et Simulation Multi-échelle, Faculté des Sciences Exactes, Département de Physique, Université Djillali Liabès de Sidi Bel Abbès 22000, Algérie*

[3]*King Abdullah University of Science and Technology (KAUST), Physical Science and Engineering Division (PSE), Thuwal 23955-6900, Saudi Arabia*

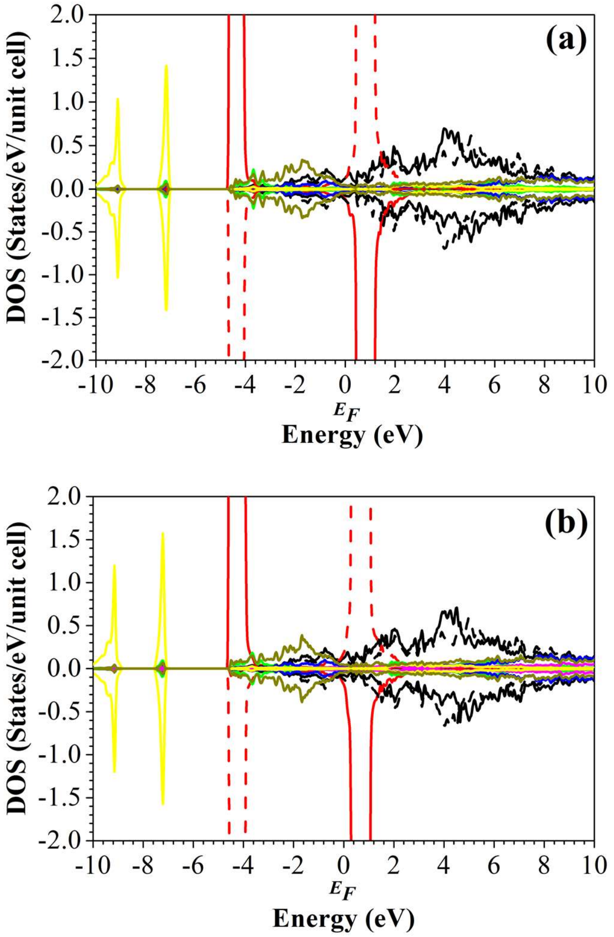

Figure S1: (a) Data of Figure 4(b) [generalized gradient approximation] compared to (b) analogous results obtained by the local density approximation.

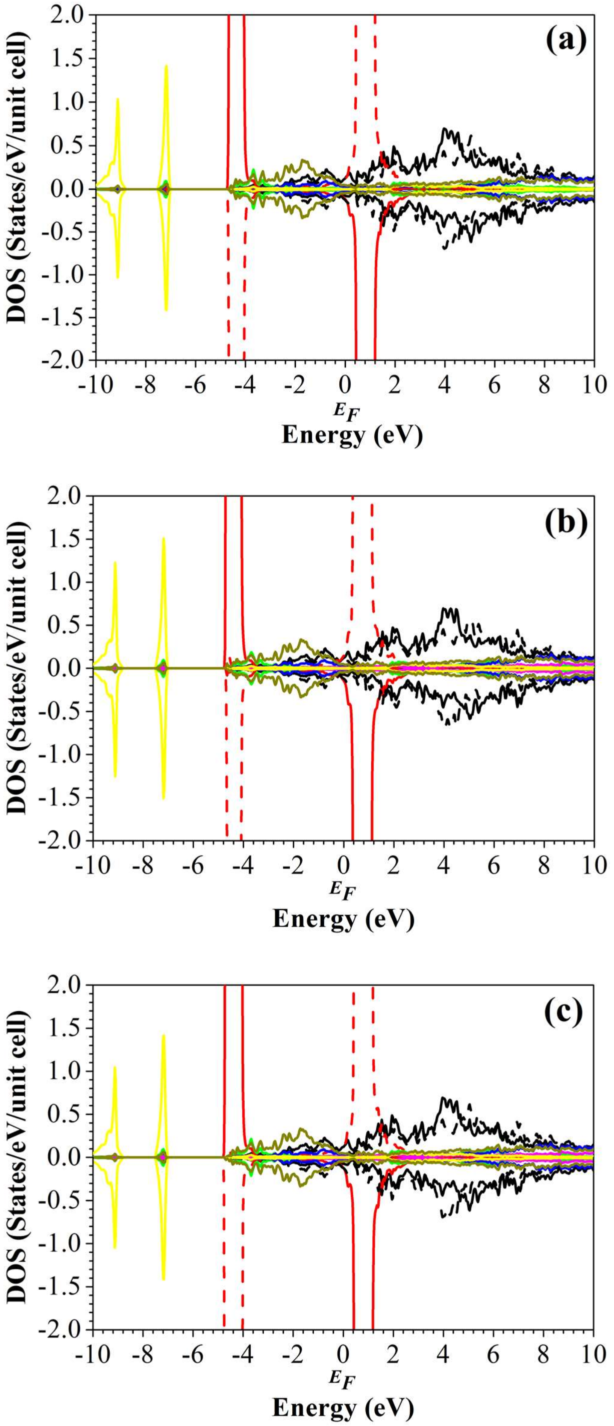

Figure S2: (a) Data of Figure 4(b) [$l_{\max} = 10$, $K_{\max} = 7/R_{\mathrm{MT,min}}$] compared to analogous results obtained for (b) $l_{\max} = 10$, $K_{\max} = 9/R_{\mathrm{MT,min}}$ and (c) $l_{\max} = 12$, $K_{\max} = 7/R_{\mathrm{MT,min}}$.
